# MRI Analysis of White Matter Myelin Water Content in Multiple Sclerosis: A Novel Approach Applied to Finding Correlates of Cortical Thinning

**DOI:** 10.3389/fnins.2017.00284

**Published:** 2017-05-26

**Authors:** Michael Dayan, Sandra M. Hurtado Rúa, Elizabeth Monohan, Kyoko Fujimoto, Sneha Pandya, Eve M. LoCastro, Tim Vartanian, Thanh D. Nguyen, Ashish Raj, Susan A. Gauthier

**Affiliations:** ^1^Department of Radiology, Weill Cornell Graduate School of Medical SciencesNew York, NY, United States; ^2^Pattern Analysis and Computer Vision, Istituto Italiano di TecnologiaGenova, Italy; ^3^Department of Mathematics, Cleveland State UniversityCleveland, OH, United States; ^4^Department of Neurology, Weill Cornell Graduate School of Medical SciencesNew York, NY, United States; ^5^Brain and Mind Institute, Weill Cornell Graduate School of Medical SciencesNew York, NY, United States

**Keywords:** multiple sclerosis, cortical thickness, white matter, myelin water fraction, RRMS, SPMS

## Abstract

A novel lesion-mask free method based on a gamma mixture model was applied to myelin water fraction (MWF) maps to estimate the association between cortical thickness and myelin content, and how it differs between relapsing-remitting (RRMS) and secondary-progressive multiple sclerosis (SPMS) groups (135 and 23 patients, respectively). It was compared to an approach based on lesion masks. The gamma mixture distribution of whole brain, white matter (WM) MWF was characterized with three variables: the mode (most frequent value) *m*_1_ of the gamma component shown to relate to lesion, the mode *m*_2_ of the component shown to be associated with normal appearing (NA) WM, and the mixing ratio (λ) between the two distributions. The lesion-mask approach relied on the mean MWF within lesion and within NAWM. A multivariate regression analysis was carried out to find the best predictors of cortical thickness for each group and for each approach. The gamma-mixture method was shown to outperform the lesion-mask approach in terms of adjusted *R*^2^, both for the RRMS and SPMS groups. The predictors of the final gamma-mixture models were found to be *m*_1_ (β = 1.56, *p* < 0.005), λ (β = −0.30, *p* < 0.0005) and age (β = −0.0031, *p* < 0.005) for the RRMS group (adjusted *R*^2^ = 0.16), and *m*_2_ (β = 4.72, *p* < 0.0005) for the SPMS group (adjusted *R*^2^ = 0.45). Further, a DICE coefficient analysis demonstrated that the lesion mask had more overlap to an ROI associated with *m*_1_, than to an ROI associated with *m*_2_ (*p* < 0.00001), and vice versa for the NAWM mask (*p* < 0.00001). These results suggest that during the relapsing phase, focal WM damage is associated with cortical thinning, yet in SPMS patients, global WM deterioration has a much stronger influence on secondary degeneration. Through these findings, we demonstrate the potential contribution of myelin loss on neuronal degeneration at different disease stages and the usefulness of our statistical reduction technique which is not affected by the typical bias associated with approaches based on lesion masks.

## 1. Introduction

The mechanisms of cortical atrophy in multiple sclerosis (MS) are poorly understood. MS is typically characterized by the appearance of white matter (WM) lesions on T2 FLAIR magnetic resonance imaging (MRI) images. Although lesions are the most visible radiological aspect of the disease, used for both diagnosis (Polman et al., 2011) and clinical trials (Polman et al., 2006; Kappos et al., 2007; Mikol et al., 2008), disability has been shown to have a stronger correlation with gray matter (GM) cortical atrophy compared to WM measurements (Ramasamy et al., 2009; Calabrese et al., 2010, 2011; Narayana et al., 2013). It is then of particular interest to explore the mechanisms driving cortical thinning.

Wallerian degeneration as a result of chronic demyelination has been implicated as one potential mechanism for cortical GM loss (Peterson et al., 2001; Vercellino et al., 2005; Geurts and Barkhof, 2008), however the extent that demyelination contributes to this process has yet to be fully elucidated. Interestingly, it has been shown that the rate of GM atrophy is markedly different between the remitting relapsing (RR) and secondary progressive (SP) phases of the disease (Fisher et al., 2008). Therefore, we hypothesize that the relationship between WM demyelination and cortical thinning is different among these two disease stages.

Quantifying WM demyelination, especially myelin loss has been challenging, and several MRI sequences have been utilized to obtain an imaging biomarker of myelin integrity in MS, including diffusion MRI (Rovaris et al., 2005), magnetization transfer ratio (MTR) imaging (Horsfield, 2005) and myelin water fraction (MWF) imaging (Laule et al., 2004). Although promising, the clinical utility of MWF maps has been impeded by a prohibitively long acquisition time of the associated T2 relaxometry MRI sequence and challenging T2 data analysis (Kolind et al., 2009). To improve the efficiency of T2 data acquisition, we optimized a 3D T2prep GRE sequence (Nguyen et al., 2012), which is able to achieve full brain coverage within 10 min at 3T and feasible to integrate into a clinical imaging protocol.

WM damage is traditionally quantified globally from the “lesion load”: the total volume of visible lesions on a dedicated MRI sequence such as FLAIR. This approach has limitations as the normal-appearing (NA) WM was shown to have pathological changes (Evangelou et al., 2000), and similarly for “dirty WM” (Seewann et al., 2009) which includes voxels with intermediate intensity between lesion and NAWM. Additionally, while advanced MRI sequences provide metrics to quantify local WM damage, they still rely on the creation of associated lesion masks. These suffer from two main issues: there is inter-rater variability in their drawing or editing, and they are time-consumming to create, thus not adapted to large cohorts (Egger et al., 2017). Automated methods for mask generation alleviate somewhat both these issues, via high reproducibility and fast computer automation, respectively. However the associated algorithms are often calibrated to generate an output in agreement with a given experimenter and therefore are inherently biased. As a result, automated methods of lesion mask creation have around the same variability between themselves as between experimenters manually drawing or editing masks.

The aim of this work was 2-fold. First, to evaluate the association between cortical thickness and WM myelin, quantified from the measured MWF distribution in whole brain WM, and assess if this relationship differed between disease stages, that is between RRMS and SPMS patients. Second, to propose for the first time a model of the distribution of cerebral white matter MWF with a statistical reduction technique which circumvents the bias introduced by the use of lesion masks.

## 2. Materials and methods

### 2.1. Subjects

Patient clinical and MRI data utilized for this analysis was part of a larger MRI and clinical database collection study at the Judith Jaffe Multiple Sclerosis Center, Weill Cornell Medicine, Department of Neurology, New York, USA. The MS database study was approved by Weill Cornell Medicine institutional review board and all subjects were consented for participation during their routine clinical visits. Informed and written consent for data collection and publication was obtained from study participants.

One-hundred and fifty-seven patients with the diagnosis of multiple sclerosis (Polman et al., 2005), including RRMS and SPMS were selected from our database. Patients were selected from our ongoing data collection, and informed consent was obtained from each subject. All RRMS and SPMS patients with a clinical MRI sequence that included the T2prep 3D spiral sequence were included in the analysis. Patient characteristics (Table 1) and clinical data were obtained within 3 months of the individual's brain MRI. The following clinical data was collected: gender, age, disease duration (DD) from initial symptom, Expanded Disability Status Score (EDSS), disease subtype, and duration of treatment with disease modifying therapy (DMT).

**Table 1:** **RRMS and SPMS group demographics and disease characteristics**.

**Characteristics**	**RRMS**	**SPMS**	**Welch two sample *t*-test**
Patients, *n*	134	23	N/A
Females	90	16	N/A
Disease duration, mean ± SD	7.7 years ± 7.3	21.7 years ± 11.0	*p* < 0.00001
Treatment Duration, mean ± SD	4.2 years ± 4.1	10.6 years ± 6.1	*p* < 0.00001
Age, mean ±SD	40.3 ± 9.7	57.4 ± 7.8	*p* < 0.00001
Cortical thickness, mean ± SD	2.46 mm ± 0.13	2.27 mm ± 0.17	*p* < 0.00001
Whole-brain WM MWF, mean ± SD	0.1388 ± 0.0205	0.1173 ± 0.0206	*p* < 0.0001

### 2.2. MRI data acquisition

Each participant underwent T1, FLAIR, and T2 relaxometry sequences with a 3Tesla GE scanner (HDxt 16.0) using 8-channel phased-array coil. The T1 sequence was an axial 3D inversion recovery fast spoiled gradient recalled echo (FSPGR) resulting in T1 volumes with 1.2 mm isotropic resolution. The T2 FLAIR volumes had 1.2 × 0.6 × 0.6 *mm*^3^ resolution. The T2 relaxometry sequence was a whole-brain T2prep 3D spiral as previously described by Nguyen et al. (2012) and included the following parameters: axial FOV = 24 cm; matrix size = 192 × 192 (interpolated to 256 × 256); slice thickness = 5 mm; number of slices = 32; sequence TR (time between subsequent T2prep pulses) = 2.5 s; spiral TR = 8.1 milliseconds (ms); spiral TE = 0.5 ms; flip angle = 10°; readout bandwidth = ±125 kHz; number of spiral leaves per segment = 64; 15 nominal T2prep times = 0, 5 ms, 10–40 ms (10 ms step), 60–140 ms (20 ms step), 180–300 ms (40 ms step); scan time = 10 min. A modified BIR-4 adiabatic pulse (De Graaf and Nicolay, 1998; Jenista et al., 2013) was used in the T2prep module to improve T2 weighting accuracy against increased B0 and B1 field inhomogeneities at 3T.

### 2.3. T2 relaxometry post-processing

**Figure 1:**
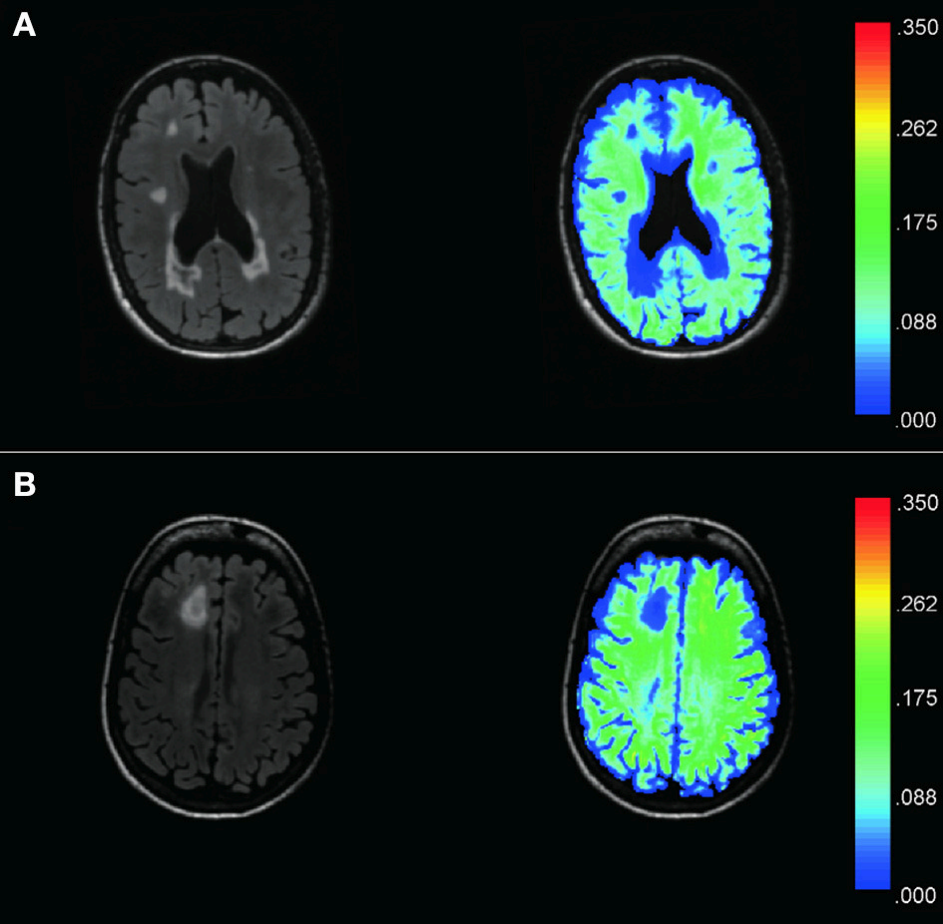
**Examples of T2 FLAIR images (left) from two MS patients (A,B)** and corresponding MWF maps (right) demonstrate good spatial resolution and sensitivity to visually T2 hyperintense lesions. The color bar represents the range of MWF values. Hyperintense lesions on T2 Flair images are found to have low MWF values as compared to the surrounding NAWM. MWF also appears sensitive to dirty WM, as shown by the low MWF displayed in dark/light blue within the central white matter of **(B)**.

Recently, we reported a multi-voxel spatial regularization approach to analyze multiexponential T2 decay data, with the goal of obtaining usable myelin maps from noisy but fast acquisitions (Kumar et al., 2012). The size and stability of the challenging minimization problem has been greatly reduced by our subsequent fitting method, called “Spatially constrained multi-Gaussian” algorithm, described in Raj et al. (2014). Briefly, it employs a non-linear model to recognize only three distinct water compartments contributing to the T2 spectrum, *s*, in each voxel: myelin water modeled by a gaussian *G*(μ_*MW*_, σ_*MW*_), intra/extra-cellular water modeld by another gaussian *G*(μ_*CW*_, σ_*CW*_) and cerebral spinal fluid modeled by a delta function δ(μ_*CSF*_). In each voxel, *s* is then given by a linear combination of these contributions: *s* = *c_MW_ G*(μ_*MW*_, σ_*MW*_) + *c_CW_ G*(μ_*CW*_, σ_*CW*_) + *c*_*CSF*_ δ(μ_*CSF*_). The MWF is defined as the ratio between the portion of the signal coming from myelin water, and the total signal from all water compartments: $MWF=\frac{c_{MW}}{c_{MW}+c_{CW}+c_{CSF}}$. The resultant MWF maps were shown to provide good spatial resolution and visual lesion discrimination (Figure 1). Note that the lesion-mask free method presented in this work and described in Section 2.6 does not depend on the method used to compute MWF.

### 2.4. T1 post-processing and cortical thickness

FreeSurfer (http://freesurfer.net) was used to segment T1 images into GM and WM masks, which were visually checked and manually edited for misclassification due to WM T1-hypointensities associated with lesions. This editing was limited to ensuring that no WM was included in the GM mask. It was different from the lesion-mask editing procedure detailed in the next section (and applied to FLAIR contrast) which involved separating NAWM from every single lesion present within the global WM mask.

After completion, a trained neurologist (SG) gave a final approval on the resulting GM and WM masks. Cortical thickness measurements were then directly obtained with FreeSurfer from the corrected masks. The 1st echo of the T2 relaxometry sequence was linearly coregistered to the subjects T1 with FSL (http://fsl.fmrib.ox.ac.uk/fsl/fslwiki). The inverse of this transform was then applied to the WM mask to get it into the same space as the MWF maps.

### 2.5. FLAIR post-processing and lesion masks

The white matter hyperintensity lesion masks were created from the T2 FLAIR images aligned onto FreeSurfer volume with the boundary-based registration (Greve and Fischl, 2009). The skullstripped FLAIR images were bias-corrected and segmented in three tissue classes with FSL FAST (Zhang et al., 2001) based on the image intensities. The third tissue class included both CSF tissue and WM lesions. Only voxels of the the bias-corrected FLAIR image belonging to that class and to the Freesurfer WM mask were kept as WM lesion candidate voxels. The associated volume was further thresholded to select only voxels with intensity greater than the 30th percentile of the FSL “robust intensity range” defined as the range of intensities between the 2nd and 98th percentile. The thresholded maps were finally binarized to provide the lesion masks.

The resulting WM lesion masks were overlaid on FLAIR images and manually edited to ensure an optimal match with the lesion hyper-intensities seen on the underlaid FLAIR contrast. A trained neurologist gave a final approval on these edits. The NAWM masks were defined as the voxels of the WM mask not belonging to the WM lesion mask. As a result the lesion and NAWM masks were mutually exclusive. The transform obtained described in the T1 post-processing section was used to obtain the lesion and NAWM masks in MWF space.

### 2.6. MWF distribution

**Figure 2:**
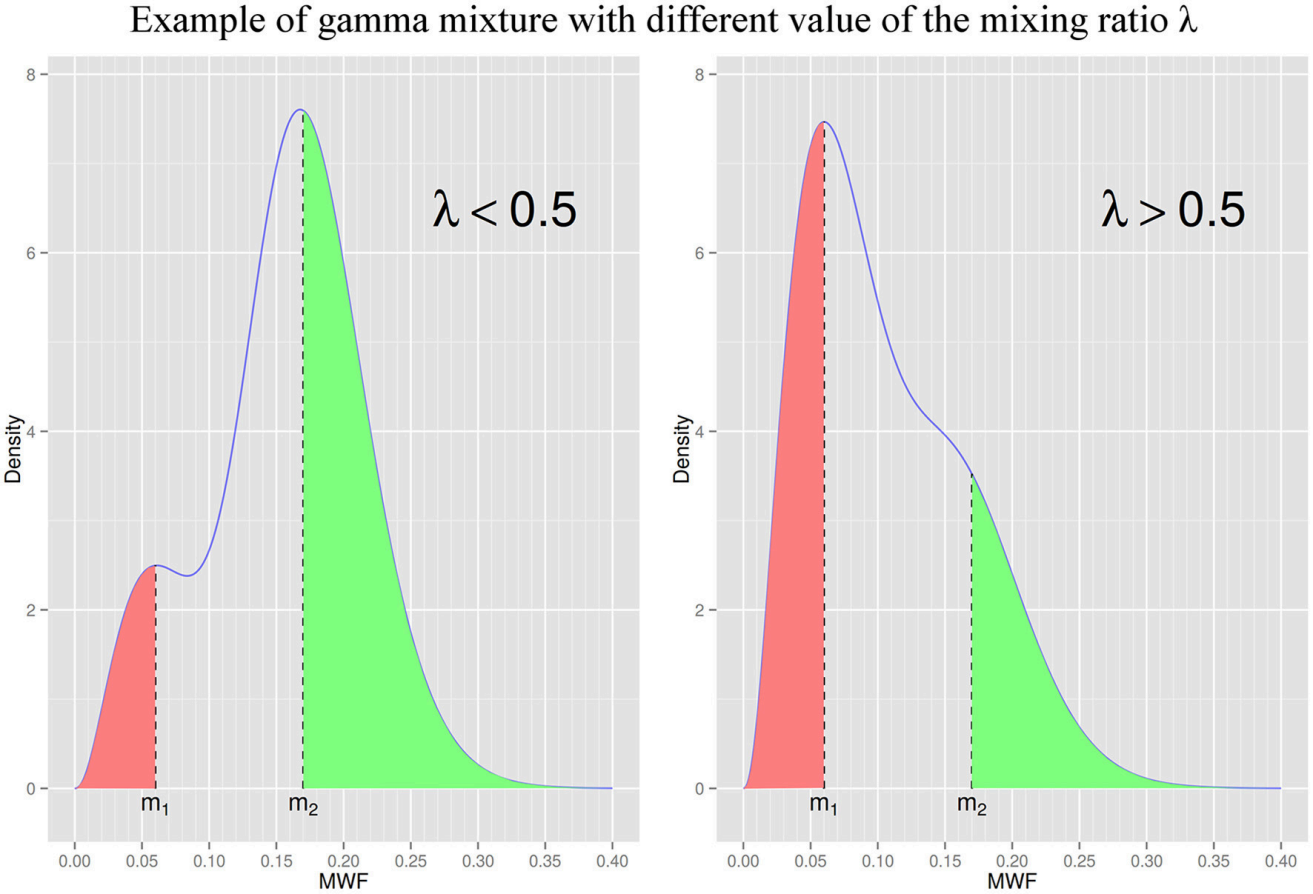
**Illustration of the same gamma mixture distribution with two different values of λ**. *m*_1_ and *m*_2_ are the modes (most frequent values) of the low and high MWF components of the gamma mixture, respectively. λ is the gamma mixture mixing ratio and represents the proportion of low MWF to high MWF (when λ is equal to 0.5, the two components have the same contribution in the gamma mixture). To compare the relationship between lesion/NAWM masks and each gamma mixture component we defined *ROI*[*m*_1_] and *ROI*[*m*_2_] as all voxels with MWF value less than *m*_1_ (in red) and greater than *m*_2_ (in green), respectively. We assumed the lesion mask had more overlap with *ROI*[*m*_1_] than *ROI*[*m*_2_], and the NAWM mask more overlap with *ROI*[*m*_2_] than *ROI*[*m*_1_]. **(Left)** Distribution with a λ value less than 0.5 (exact parameters: *m*_1_ = 0.06, *m*_2_ = 0.17, λ = 0.25). **(Right)** Distribution with a λ value greater than 0.5 (exact parameters: *m*_1_ = 0.06, *m*_2_ = 0.17, λ = 0.75).

**Figure 3:**
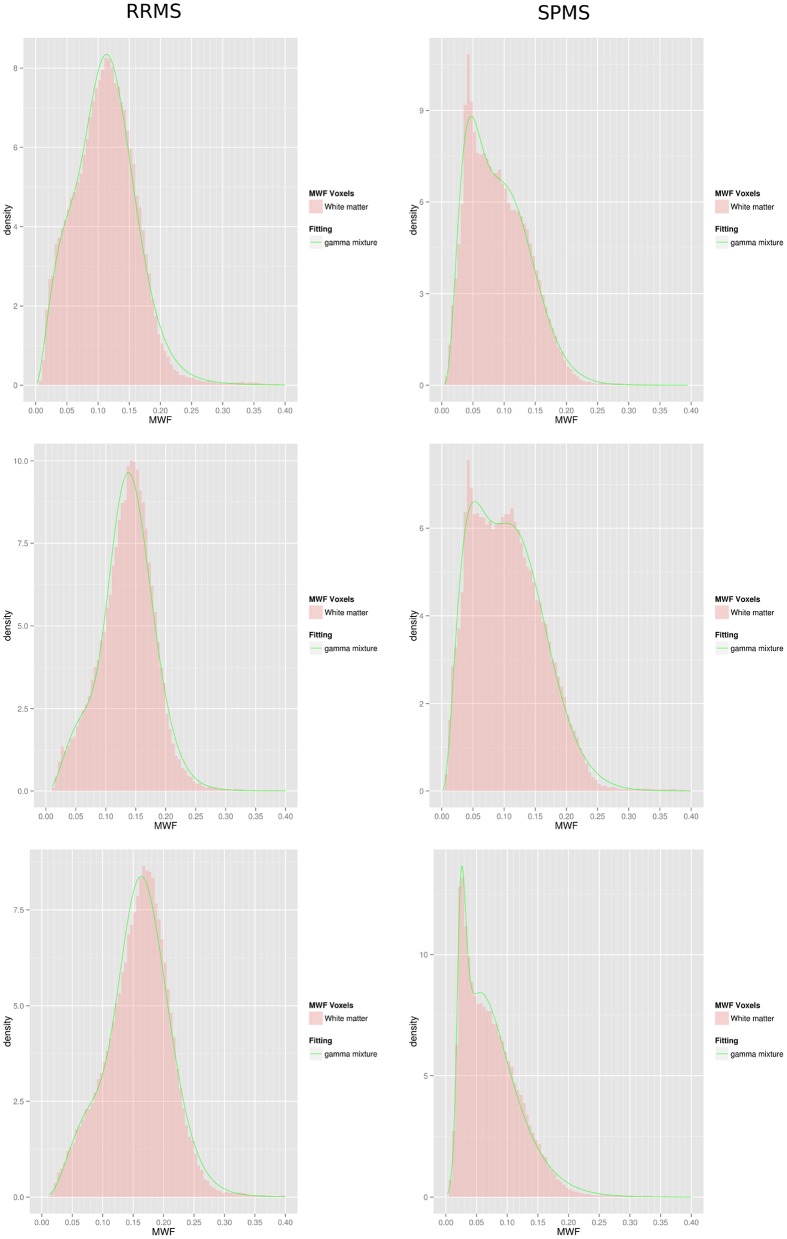
**Gamma mixture fits of 3 RRMS subjects (left column)** and 3 SPMS subjects **(right column)**. Histograms are only represented for illustrative purposes and were not used during the fitting procedure.

We analyzed MWF maps within WM masks for each patient using plots and descriptive statistics. The observed distributions of MWF values (at the patient level) appeared skewed and with one or two prominent peaks (Figures 2, 3). One of the most common skewed distribution is the gamma distribution, used in fields ranging from economics (Chatterjee and Chakrabarti, 2007; Chotikapanich and Griffiths, 2008) to meteorology (Smith, 2003; Yoo et al., 2005) as either a single distribution (Smith, 2003; Chatterjee and Chakrabarti, 2007) or mixture of two distributions (Yoo et al., 2005; Chotikapanich and Griffiths, 2008). The skewed distribution of our data also presented one or two peaks and exploratory analysis demonstrated a tight visual fit from a gamma model. These elements provided ground for describing the data distributions as either a gamma random variable or a mixture of two gamma-distributed random variables. In other words, we hypothesized that for each patient *i*, the MWF distribution *p*_*i*_(*MWF*) followed a mixture of two gamma distributions of the form *p*_*i*_(*MWF*) = λ_*i*_Γ(*MWF*; α_1, *i*_, β_1, *i*_)+(1−λ_*i*_)Γ(*MWF*; α_2, *i*_, β_2, *i*_) where Γ denotes a gamma distribution, λ_*i*_ is the proportion of mixing between the two distributions, and the “shape” and “scale” pairs (α_1, *i*_, β_1, *i*_) and (α_2, *i*_, β_2, *i*_) are the parameters describing the shape and scale of each mixture component (Figure 2). In contrast, using simply the mean MWF would have lost the information contained in this distribution, especially the presence of two peaks.

The shape parameter controls the skewness of the distribution while the scale controls how spread the distribution is. The position of the peak in each component of the distribution, i.e., the distribution modes, are given by *m*_1_ = (α_1_−1) × β_1_ and *m*_2_ = (α_2_−1) × β_2_. Another reason we chose the gamma distribution as opposed to another one, is that the parameters are easier to interpret, and directly relate to explicit quantities such as the mean and modes of the distribution as just described. For the single gamma distribution, parameters were estimated with the maximum-likelihood (ML) method, while for the gamma mixture distribution they were estimated with the expectation maximization (EM) method (Dempster et al., 1977). A penalized likelihood ratio test was implemented to determine the goodness of fit of the mixture gamma compared to the single gamma model (Wong and Li, 2014) and choose between the two.

### 2.7. Interpretation of the gamma mixture parameters in terms of lesion and NAWM

Our assumption was that the lesion mask was more associated to the low-MWF component at position *m*_1_, compared to the high-MWF component at position *m*_2_. Similarly, we expected the NAWM mask to be more associated to the high-MWF component than to the low-MWF component. To test the first assumption, we computed a metric of similarity between the lesion mask and a region of interest *ROI*[*m*_1_] associated with the low-MWF component at *m*_1_, and between the lesion mask and a region *ROI*[*m*_2_] associated with the high-MWF component at *m*_2_. We then tested if the metric of similarity was significantly higher between the lesion mask and *ROI*[*m*_1_] than between the lesion mask and *ROI*[*m*_2_]. The metric of similarity was chosen to be the Dice coefficient, commonly use in neuroimaging to compute the overlap of two ROIs (see e.g., Dayan et al., 2015). It is defined for two regions A and B as $Dice\left(A,B\right)=\frac{2\times |A\cap B|}{\left|A|+|B\right|}$ where |*X*| stands for the number of voxels in region *X*. *ROI*[*m*_1_] was defined as the region with voxels having MWF value below *m*_1_, and *ROI*[*m*_2_] was defined as the region with voxels having MWF value above *m*_2_ (Figure 2). We therefore tested if *Dice*(lesion mask, *ROI*[*m*_1_]) > *Dice*(lesion mask, *ROI*[*m*_2_]), using a Welch two sample *t*-test. Similarly, to test our second assumption that the NAWM mask is more associated to the high-MWF component than to the low-MWF component, we used a Welch two sample *t*-test to test that *Dice*(NAWM mask, *ROI*[*m*_2_]) > *Dice*(NAWM mask, *ROI*[*m*_1_]).

### 2.8. Group analysis

To assess the association between the distribution of MWF for our cohort of MS patients and cortical thickness, we used a multivariate linear regression model. In order to compare our statistical reduction technique with a classical method based on lesion masks, we used two sets of possible predictors. For our gamma-mixture approach we considered as predictor variables the MWF distribution mixing factor λ, and distribution modes *m*_1_ and *m*_2_. For the standard approach based on lesion masks, we used mean MWF in the lesion mask validated by the trained neurologist, and mean MWF in the mutually exclusive NAWM mask. We also considered for both sets of predictors age, gender, DMT and DD. The best model for each approach and each group was chosen according to the procedure detailed in the Appendix.

One aim was to compare the best model obtained with predictors derived from our statistical reduction method and the best model obtained with predictors derived from the classical approach based on lesion masks. This was carried out in each group by comparing the adjusted *R*^2^ of these two models. Another aim was to examine if the final models were different in the RRMS and SPMS group, and interpret the models in terms of their predictors. To help in this endeavor, we assessed the contribution of each coefficient to *R*^2^ by calculating the average contribution of that coefficient when added to all possible models excluding that coefficient, as in Lindeman et al. (1980).

All *p*-values were two-sided with statistical significance evaluated at the 0.05 alpha level. Statistical analysis was conducted using R: A Language and Environment for Statistical Computing, R Development Core Team, Vienna, Austria, 2011, http://www.R-project.org.

## 3. Results

### 3.1. Multiple metrics measured in a large cohort of RRMS and SPMS patients

In all patients, the T2 spiral sequence was easily placed within the clinical protocol given the feasible acquisition time of only 10 min. The combination of T2 spiral acquisition and multi-Gaussian post-processing algorithm produced whole brain, high-resolution MWF white matter maps with good lesion detection (Figure 1).

Cortical thickness measures and other patient characteristics in each of the RRMS and SPMS groups are summarized in Table 1. Univariate *t*-tests (Welch Two Sample *t*-tests) demonstrated significant differences in age, cortical thickness and mean whole-brain WM MWF between the RRMS and SPMS groups.

### 3.2. Modeling whole brain white matter MWF distribution

Lesion centric data analysis based upon semi-automated lesion mapping programs introduces a bias based upon the reviewer's determination of perceived regions of pathology. We know that regions considered to be dirty WM on T2 imaging have less myelin (Moore et al., 2008; Seewann et al., 2009), therefore we considered an unbiased approach to model each individual's whole brain white matter MWF data as either a gamma random variable or a mixture of two gamma-distributed random variables. The likelihood ratio test provided *p*-values less than 0.00001 in all patients, thus there was statistical evidence to conclude that a gamma mixture distribution was preferred over the single gamma distribution. As a result the gamma mixture model was chosen for all patients. Further, a univariate *t*-test demonstrated a significant difference in mean whole-brain WM MWF between the RRMS and SPMS groups (Table 1), suggesting a possible differential distribution of MWF in each of these groups. Figure 3 includes examples of 6 individual patients; the figure shows a histogram of the MWF raw data and the smooth green curve represents the fitted mixture distribution. Different distribution shapes according to MS groups are visible on that figure, with a trend to higher *m*_1_ and *m*_2_, and lower λ, in RRMS patients compared to SPMS patients. Note that the method used to carry out the gamma mixture fitting, expectation maximization, did not rely on the binning of the data or its representation as an histogram. Histograms have been created exclusively for visualization of the fitting procedure outcome.

### 3.3. Relationship between gamma mixture components and lesion/NAWM masks

**Figure 4:**
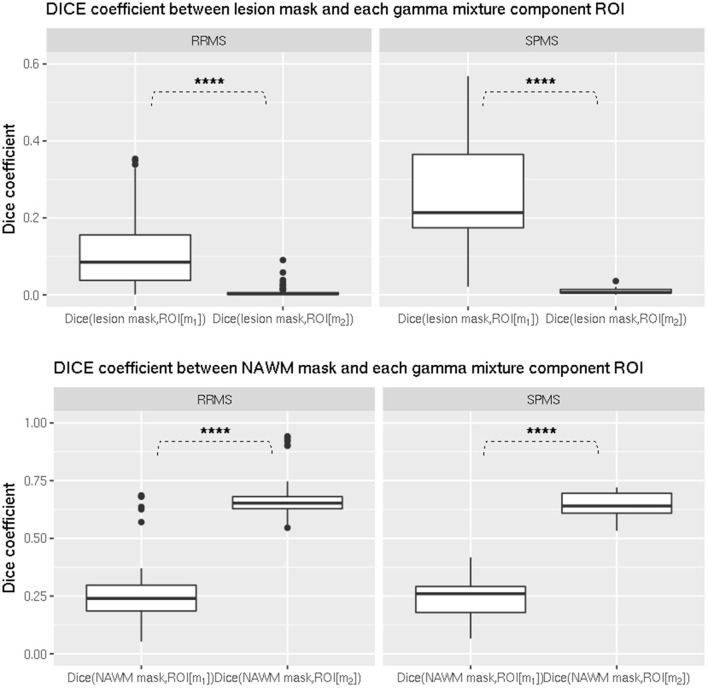
**Comparison of dice coefficient between ROIs from gamma mixture components and lesion/NAWM masks in (left) RRMS and (right) SPMS groups. (Top)** Comparison between lesion mask and *ROI*[*m*_1_], and lesion mask and *ROI*[*m*_2_] in (left) RRMS and (right) SPMS groups. The difference was shown to be significant in both RRMS (*p* < 0.00001) and SPMS (*p* < 0.00001) groups. **(Bottom)** Comparison between NAWM mask and *ROI*[*m*_1_], and NAWM mask and *ROI*[*m*_2_] in (left) RRMS and (right) SPMS groups. The difference was shown to be significant in both RRMS (*p* < 0.00001) and SPMS (*p* < 0.00001) groups. *p < 0.01, **p < 0.001, ***p < 0.0001, ****p < 0.00001.

To interpret the gamma-mixture model parameters in terms of the underlying pathology, we tested that the lesion mask had a higher Dice coefficient with *ROI*[*m*_1_], defined from the first gamma component, than with *ROI*[*m*_2_], defined from the second component, in both RRMS and SPMS groups. The *t*-test demonstrated it was significantly higher in each group (*p* < 0.00001, Figure 4, top).

Similarly we tested that the NAWM had a higher Dice coefficient with *ROI*[*m*_2_] than with *ROI*[*m*_1_] in both groups, and it was also significantly higher in each group (*p* < 0.00001, Figure 4, bottom).

**Figure 5:**
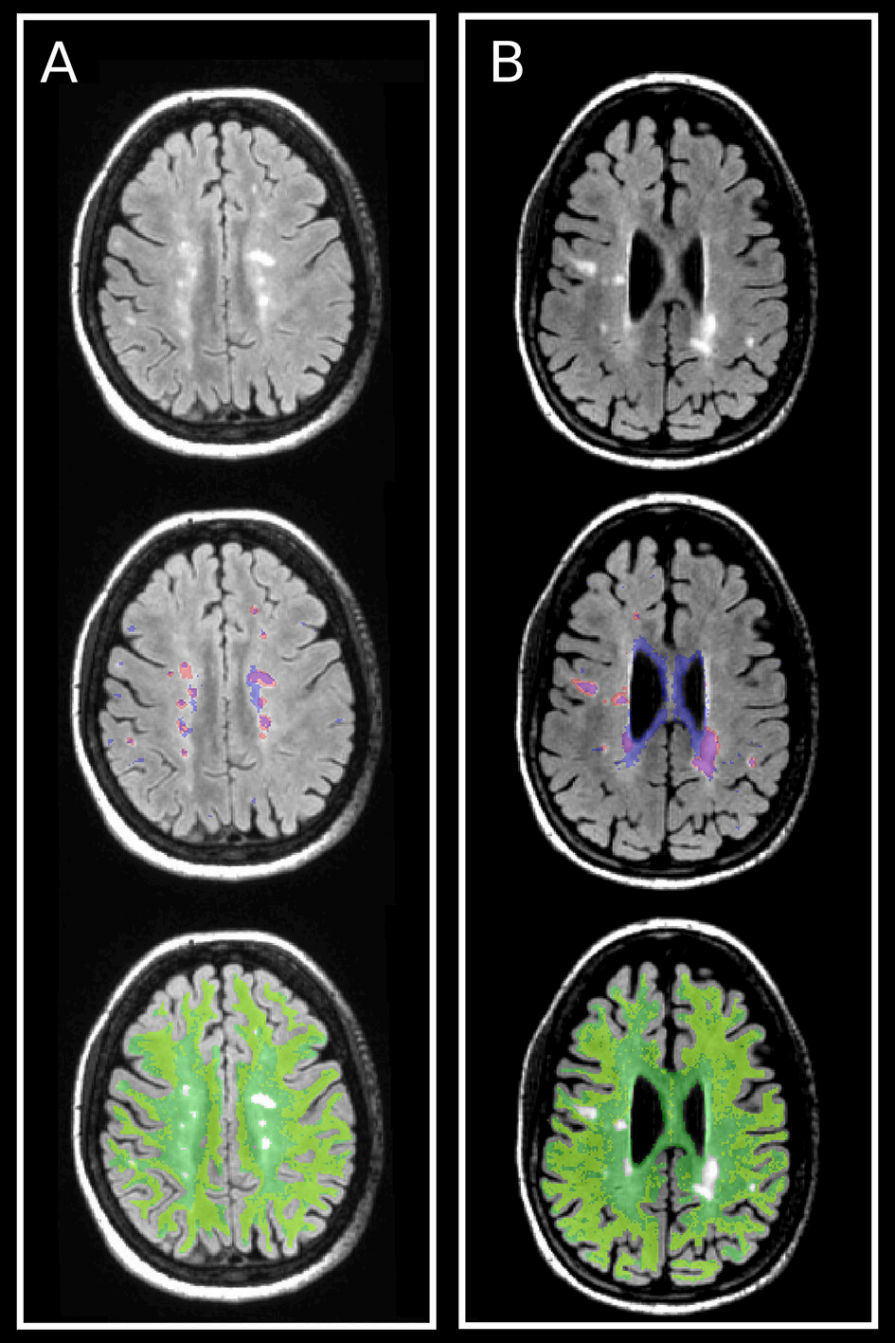
**Illustration of lesion and NAWM masks, and their relationships with the ROIs defined from the gamma mixture, ***ROI***[***m***_**1**_] and ***ROI***[***m***_**2**_], in one (A)** RRMS and one **(B)** SPMS patients. (Top) FLAIR images with hyperintensities corresponding to lesions. (Middle) Lesion mask (red) and *ROI*[*m*_1_] (blue). (Bottom) NAWM mask (green) and *ROI*[*m*_2_] (yellow). *ROI*[*m*_1_] and *ROI*[*m*_2_] have been created from the gamma mixture so that not to overlap one with the other (cf. Figure 2), and as a result they only represent part (approximately half) of each gamma component. Despite this limitation, it can be clearly seen that the lesion mask is more similar to *ROI*[*m*_1_] than to *ROI*[*m*_2_], and that the NAWM mask is more similar to *ROI*[*m*_2_] than to *ROI*[*m*_1_], as corroborated by the DICE coefficient calculations shown in Figure 4.

This provided support for interpretating *m*_1_ as associated to lesioned WM and *m*_2_ to NAWM. An illustration of lesion and NAWM masks, as well as *ROI*[*m*_1_] and *ROI*[*m*_2_], is provided in Figure 5. It can be seen that *ROI*[*m*_1_] tend to overlap with the lesion mask while also including dirty WM, while *ROI*[*m*_2_] is mostly included within NAWM and tend to exclude lesion and dirty WM.

### 3.4. Predictors of cortical thickness for SPMS and RRMS patients

We reasoned that the degree of cortical atrophy may directly correlate with whole brain myelin content. More precisely, we wanted to test the hypothesis that MWF distribution was associated with cortical thickness, and that a differential association existed between MS groups. We also investigated how a fully automated model performed to accomplish this objective compared to a classical approach based on manually edited lesion masks.

For the gamma-mixture approach, we utilized three metrics from the MWF distribution (λ, *m*_1_, *m*_2_) to explore the relationship of myelin and cortical thickness among RRMS and SPMS patients. For the standard approach we used the mean MWF in the lesion mask and the mean MWF in the NAWM mask. In both cases we controlled for other demographics and patient predictors, (age, gender, DD, DMT) and applied an exhaustive model search coupled to 10-fold cross-validation to select the best model for each approach and for each RRMS and SPMS group (cf. Appendix). For the classical approach, the final parameters kept in the model were {age, mean lesion MWF} with a resulting adjusted *R*^2^ of 0.13 for the RRMS group, and were solely {mean NAWM MWF} with a resulting adjusted *R*^2^ of 0.39 for the SPMS group. For the statistical reduction approach, the final parameters were {λ, *m*_1_, *age*} with an adjusted *R*^2^ of 0.16 for the RRMS group, and were {*m*_2_} with an adjusted *R*^2^ of 0.45 for the SPMS group. The greater adjusted *R*^2^ for the gamma-mixture approach demonstrated that the automated method outperformed the lesion mask approach to explain the variance in cortical thickness in either patient group. Further, comparing the final models in each approach, {mean NAWM MWF} and {*m*_2_} for the SPMS group, and {age, mean lesion MWF} and {λ, *m*_1_, *age*} for the RRMS group, brought additional evidence of the surrogate relationship between NAWM and the second gamma-mixture component, and lesion and the first gamma-mixture component.

Analyzing in more details the statistical reduction model, we found that for the RRMS group, the regression coefficients of the predictor variables were −0.0031 (*p* < 0.005) for age (in years), 1.56 (*p* < 0.005) for *m*_1_ (in MWF, a ratio) and −0.30 (*p* < 0.0005) for λ (a ratio). One can interpret these coefficients as showing that on average a cortical thickness decrease of 0.05 mm is associated with an age increase of 16 years (all other variables held constant), an *m*_1_ decrease of 0.032 (all other variables held constant) or a λ increase of 0.17 (all other variables held constant). As a reminder, *m*_1_ and *m*_2_ are the two modes of the gamma mixture distribution, corresponding to the low and high MWF components of the distribution, determined to relate to lesion and NAWM, respectively, while λ is the mixing ratio determining the proportion of each component in the mixture. A high λ represents a higher contribution of low WM MWF to the gamma mixture while a low λ represents a higher contribution of high WM MWF (Figure 2).

**Figure 6:**
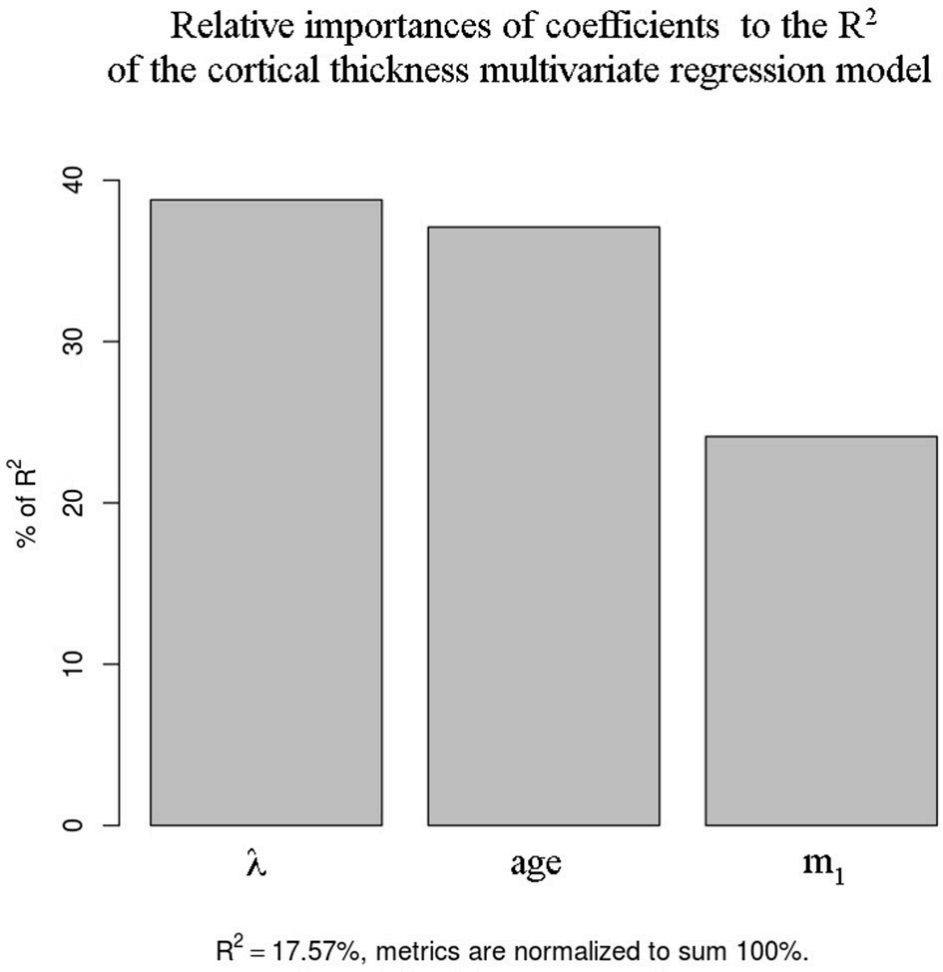
**Relative contribution to total ***R***^**2**^ of the cortical thickness regression coefficients for the RRMS group**.

The contribution of each coefficient to the absolute *R*^2^ is shown in Figure 6. It can be seen that in decreasing order (in terms of contribution to *R*^2^) the coefficients are λ, age and *m*_1_, all having a relative importance greater than 20%.

**Figure 7:**
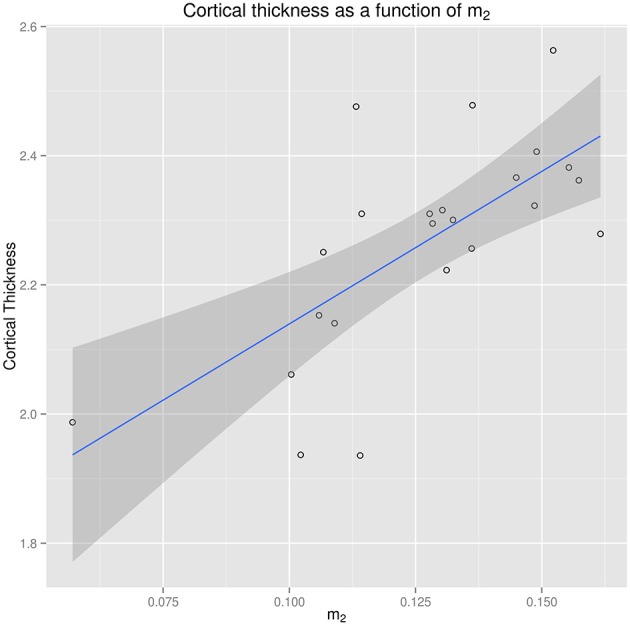
**Regression of cortical thickness from the position of the gamma mixture second mode ***m***_**2**_ in the SPMS group**.

For the SPMS group, the regression coefficient was 4.72 (*p* < 0.0005) for *m*_2_, i.e., on average, a cortical thickness decrease of 0.05 mm is associated with an *m*_2_ decrease of 0.011. The association between cortical thickness and the position of the gamma mixture second mode *m*_2_ is illustrated in Figure 7.

These results suggest a marked difference in the association between cortical thickness and WM MWF distribution in RRMS and SPMS patients.

## 4. Discussion

Progressive disability in MS is related to cortical thinning (Calabrese et al., 2007; Ceccarelli et al., 2008; Ramasamy et al., 2009; Roosendaal et al., 2011; Batista et al., 2012) and thus important insights into MS could be obtained from understanding the mechanisms driving this cortical loss. The cause of cortical GM atrophy, and whole brain GM damage in general, in multiple sclerosis is currently unclear. Although correlation between WM insult (in terms of lesion load) and GM atrophy has been shown in a number of studies, other investigations support other independent processes, such as inflammation. We hypothesized that these mechanisms differ according to the stage of the disease, notably WM pathology which is the focus of the present study. The statistical analysis undertaken allowed to explore the differential WM correlates of cortical thinning in RRMS and SPMS patients while avoiding the bias introduced by lesion masks.

### 4.1. Gamma-mixture fitting applied to MWF

Multi-component T2 relaxometry (Koenig et al., 1990; MacKay et al., 1994, 2006; Whittall et al., 1997; Laule et al., 2004, 2007) is a promising noninvasive method to measure MWF. MWF has been shown to correlate highly with histological myelin measurement in rodents (Gareau et al., 2000; Kozlowski et al., 2008; McCreary et al., 2009) and myelin content in post-mortem MS samples, (Moore et al., 2000, 2008; Laule et al., 2006, 2008, 2011) but there have been limited studies translating this technique to study the association with MS disease variables. Through the clinical feasibility of our 10-min whole brain T2prep 3D spiral sequence, we were able to apply this technology to a large cohort of multiple sclerosis patients. Note that although we chose MWF for this study, any surrogate marker of myelin could be used with our novel statistical reduction technique, such as magnetization transfer ratio (Horsfield, 2005) or diffusion MRI metrics (Rovaris et al., 2005).

We illustrated the use of our whole brain white matter gamma-mixture fitting on some of the largest MWF quantitative analyses conducted on multiple sclerosis patients. Another hurdle when investigating WM damage is localizing abnormal WM. Semi-automated delineation of WM lesions has been the gold standard for this purpose although it requires expert knowledge, is particularly time consuming, and biased in nature. We proposed an alternative solution by examining all WM voxels without the use of user-defined lesion masks. Whole brain approaches have been carried out previously with histogram analyses, both from MTR and diffusion MRI data (see for example Van Buchem et al., 1996; Cercignani et al., 2001, respectively). In the current study we chose a whole WM approach which, contrarily to the aforementioned studies, preserves an interpretation of the model parameters in terms of damaged and NAWM. The visible skewness of the data and the possible presence of two peaks led to choose between a single gamma and a gamma mixture, with the latter demonstrated to provide the best fit in all subjects. The position of the peaks of the first and second components were shown to relate to the amount of pathological and NAWM, respectively, as shown by the significant difference in DICE coefficients. Beyond lesional WM, the first component is assumed to also include dirty WM with MWF values lower than the high MWF component. This results in larger *ROI*[*m*_1_] volume compared to lesion masks, and would explain why the absolute value of *Dice*(lesion mask, *ROI*[*m*_1_]) is not as high as between *Dice*(NAWM, *ROI*[*m*_2_]): the relatively limited size |lesion mask| of the lesion volume constrains the Dice numerator |lesion mask∩*ROI*[*m*_1_]|, and thus the Dice coefficient itself, not to be large. Explicitely and automatically distinguishing between NAWM, with high MWF, and lesional and dirty WM, with low MWF, is a strength of the gamma mixture model.

The process of biomarker development requires qualification, i.e., linking the biomarker to the biological process and clinical endpoints. This work aimed at qualifying MWF by demonstrating its relevance to explain some of the variance in patient cortical thickness and to differentiate between disease subtypes, namely RRMS and SPMS. Through our statistical approach, three variables describing the distribution of each patient's whole brain MWF (*m*_1_, *m*_2_, λ) were used as independent variables for the multi-variate analysis. Importantly, the distributions were created for each individual patient without a prior classification of the data; specifically we did not identify lesion or NAWM. This approach removes a potential bias introduced into lesion identification through manual delineation or correction if lesion-detection is partly automated. We compared it with models based on mean lesional and NAWM calculated from lesion masks, and found the adjusted *R*^2^ of the gamma-mixture models to be higher. This demonstrated the superiority of our statistical reduction technique despite bypassing the time-consumming step of expert drawing of lesion masks. Importantly, the modeled mixture parameters could be interpreted in terms of the underlying WM aspects. Variation in its lowest mode *m*_1_ corresponds to changes in abnormal WM, variation in *m*_2_ corresponds to changes in NAWM and λ indicates the proportion of abnormal to NAWM.

### 4.2. Differential relationship of cortical thinning between RRMS and SPMS groups as assessed from the gamma mixture model

The multivariate regression models for RRMS and SPMS demonstrated that the MWF model parameters (*m*_1_, *m*_2_, λ) were significant in explaining the variance in cortical thickness, but formed complementary subsets of predictors depending on the group at stake: λ and *m*_1_ (together with age) were significant predictors for RRMS patients while only *m*_2_ was a significant predictor for SPMS patients. This suggests that different processes are ongoing, or similar processes but with different magnitudes, during these two stages of the disease. The variables *m*_1_ and λ are associated with low MWF, assumed to represent pathological WM, and the fact we did not find them to be significant predictors of cortical GM atrophy in the SPMS group contrarily to the RRMS group is an interesting finding. This is in line with the recent work of Steenwijk et al. (2014) and Steenwijk et al. (2015) who similarly reported a differential relationship of WM damage and cortical loss among RRMS and SPMS patients. In this study traditional T2 hyperintense lesion load characterized WM pathology. It was shown to be a significant predictor of cortical thickness in the RRMS group but not in the SPMS group, for which no WM correlate of cortical thinning was found (Steenwijk et al., 2014). The advantage of our study was the utilization of an MRI metric more specific to myelin damage, namely MWF, with which we could more accurately characterize this relationship. It notably led us to find a surrogate of NAWM, *m*_2_, highly correlated with cortical thinning in the SPMS group, and provided strong support for the existence of a differential relationship of cortical thinning between RRMS and SPMS groups.

The moderate adjusted *R*^2^ (*R*^2^ = 0.16) obtained for the RRMS group to explain cortical thickness suggests that an increase of abnormal WM in terms of relative amount and pathology (as indicated by λ and *m*_1_, respectively) is associated to cortical thinning, although in a limited fashion. More precisely, a change in *m*_1_ is assumed to result from focal demyelination, likely predominantly found within lesions and dirty WM. These results suggest that a small contribution of secondary degeneration is related to focal WM myelin injury and other sources not included in our model such as normal aging and primary disease related GM pathology (i.e., GM lesions Seewann et al., 2011), may drive the decrease in cortical thickness. The comparatively high *R*^2^ calculated in the SPMS group with only a predictor corresponding to NAWM suggests that cortical thinning has little dependence on focal WM damage (of which *m*_1_ is a surrogate marker). In contrast to RRMS, WM with higher MWF, associated to *m*_2_ and often reprensenting the larger volume of WM, had a profound effect. It may be related to more diffuse inflammation at this stage of the disease and consequently to more global white matter damage (Kutzelnigg et al., 2005). Interestingly, as in Steenwijk et al. (2014), we did not find any influence of age on cortical thinning in SPMS patients, which suggests that age-related neuronal loss is likely outweighed by a shift of NAWM toward lower MWF, possibly illustrating overwhelming global white matter damage.

### 4.3. Limitations

The causality relationship between WM demyelination and cortical thinning is restricted to an assumption in this work, and a longitudinal study would be required to validate it. Additionally we investigated global GM atrophy, and did not refine the analysis in terms of individual GM regions. Both of these aspects will be tackled in future research using the profilometry framework we introduced recently in Dayan et al. (2016). It would be particularly useful for these two aims, as it can identify WM tract connecting a given pair of GM regions, and assess how MWF changes with time along these tracts. This approach would be advantageous not only in diseases involving whole brain WM, such as in MS, but also in disorders known to affect markedly specific WM tracts, such as the cerebellar peduncles in ataxia (Dayan et al., 2017) and the corticospinal tracts in amyotrophic lateral sclerosis (Turner et al., 2009). It will be combined with Kuceyeski et al. (2015) to explore the association between GM/WM damage and clinical scores, which was not done in this work. Another limitation is the lack of a cohort of normal controls to further interpret the model parameters. An effort dedicated to recruiting healthy subjects has been started to remedy this impediment. Finally, although we circumvented the creation of lesion masks and its associated bias, part of the processing pipeline still required some minimal editing of the border between GM and WM masks which could be affected by lesions.

## 5. Conclusion

The relationship between cortical GM loss and WM damage in MS is important to elucidate. We presented in this work a whole brain WM model to investigate this relationship at the RRMS and SPMS stage of the disease avoiding the use of lesion masks and their associated bias. Our results represent one of the first quantitative studies characterizing the distribution of whole brain WM MWF within a large cohort of MS patients and relating these measures to cortical atrophy.

Our findings supports the idea that secondary degeneration could play a role in cortical thinning during the first phase of MS as a result of focal demyelination, in parallel with other processes such as GM lesions. We also showed that a different mechanism affected SPMS patients, where high MWF WM presented a strong correlation with cortical GM loss, possibly driving this atrophy. A longitudinal analysis would be required to ascertain that cortical thinning appears after WM damage.

The data reduction technique presented was shown to unravel a variety of different relationships between WM and GM while collapsing the high dimensionality of MWF maps to a few parameters. As it does not rely on lesion masks, it avoids the associated experimenter bias and does not suffer from the potential time-consumming operations required to create such masks. These are important advantages which should make this technique suitable for other MS studies.

Our study suggests that MWF, as an indirect biomarker of myelin, is a promising tool to study the influence of myelin change on cortical atrophy. The rate of cortical loss has been suggested to relate to the rate of disease progression and our results provide further evidence that inducing remyelination would also function as a neuroprotective therapeutic strategy (Piaton et al., 2010). Coupled to our gamma distribution model, this imaging biomarker could help adapt therapeutics according to the different disease stages. In particular, intervention early on would ideally prevent conversion to the progressive stage of the disease. To this aim, future work will include examining the variability of these results across MWF calculation algorithms and MRI scanners.

## Author contributions

MD participated in the design, statistical modeling, data analysis, interpretation and writing of the work. SH participated in the design, statistical modeling, data analysis, interpretation and editing of the manuscript. KF, SP, EL, and EM participated in the data analysis and revising of the manuscript. TV participated in design and revising of the manuscript. TN and AR participated in the design, data analysis and revising of the manuscript. SG contributed as senior author and participated in the design, data analysis and editing of the manuscript.

### Conflict of interest statement

TV is a speaker for Teva Neuroscience and Mallinckrodt, has received honoraria for advising Genzyme, and has received grant support from the National MS society, Biogen, and Mallinckrodt. SG has received honoraria for advising Genentech and Teva Neuroscience and has received grant support from the National MS society, Biogen, Novartis Pharmaceuticals, Mallinckrodt, Genzyme, and EMD Serono. The other authors declare that the research was conducted in the absence of any commercial or financial relationships that could be construed as a potential conflict of interest.

## Appendix

### Selection of the best multivariate linear regression model

For each approach and each group of MS patients, we had to choose the best model with respect to the N predictors available. This was implemented in two steps.

First, given a group and a number K of predictors, we did an exhaustive search to choose the best K-predictor model. This was done by comparing all possible K-predictor models, and selecting the one with the minimum residual sum of squares (RSS).

Second, among the best 1-predictor, 2-predictor, …, N-predictor models, we used 10-fold cross-validation to select the one with the minimum test-RSS. Briefly, the data is split in 10 subsets (“folds”), and selecting 1-fold in turn, the model is fitted on the remaining 9 folds before being tested on the selected fold. The test-RSS is the average RSS obtained from this iterative procedure on the 10 folds: test-RSS = mean(test-RSS_fold1_, test-RSS_fold2_, …test-RSS_fold10_).
